# Dimethyl 3,3′-[(4,5-dicyano-1,2-phenyl­ene)bis­(­oxy)]dibenzoate

**DOI:** 10.1107/S1600536812027432

**Published:** 2012-08-01

**Authors:** Ming Bai, Yan Zhang, Chongxi Zhang

**Affiliations:** a Marine College, Shandong University at Weihai, Weihai 264209, People’s Republic of China

## Abstract

In the title compound, C_24_H_16_N_2_O_6_, the dihedral angles between the central 4,5-dicyano-1,2-phenyl­ene unit [maximum deviation from planarity = 0.014 (4) Å] and the pendant benzene rings are 73.62 (5) and 84.08 (6)°.

## Related literature

For background to the properties and applications of phthalocyanines, see: Jiang & Ng (2009 ▶); Wang *et al.* (2011 ▶). For the synthesis, see Wang *et al.* (2009 ▶).
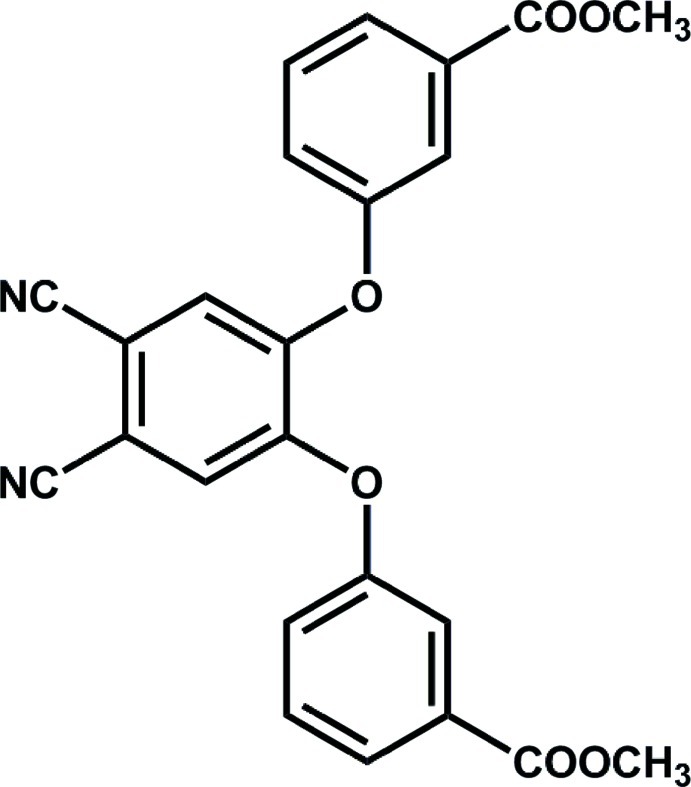


## Experimental

### 

#### Crystal data

C_24_H_16_N_2_O_6_
*M* *_r_* = 428.39
Triclinic, 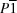

*a* = 10.1092 (11) Å
*b* = 10.3408 (11) Å
*c* = 10.8190 (14) Å
α = 82.284 (10)°
β = 85.991 (10)°
γ = 64.721 (11)°
*V* = 1013.3 (2) Å^3^
*Z* = 2
Cu *K*α radiation
μ = 0.86 mm^−1^
*T* = 293 K
0.15 × 0.11 × 0.08 mm

#### Data collection

Agilent Xcalibur Eos Gemini diffractometer
Absorption correction: multi-scan (*CrysAlis PRO*; Agilent, 2011 ▶) *T*_min_ = 0.882, *T*_max_ = 0.935
6668 measured reflections
3484 independent reflections
2790 reflections with *I* > 2σ(*I*)
*R*_int_ = 0.019

#### Refinement

*R*[*F* ^2^ > 2σ(*F* ^2^)] = 0.039
*wR*(*F* ^2^) = 0.119
*S* = 1.07
3484 reflections
292 parameters
1 restraint
H-atom parameters constrained
Δρ_max_ = 0.21 e Å^−3^
Δρ_min_ = −0.20 e Å^−3^

### 

Data collection: *CrysAlis PRO* (Agilent, 2011 ▶); cell refinement: *CrysAlis PRO*; data reduction: *CrysAlis PRO*; program(s) used to solve structure: *SHELXS97* (Sheldrick, 2008 ▶); program(s) used to refine structure: *SHELXL97* (Sheldrick, 2008 ▶); molecular graphics: *XP* in *SHELXTL* (Sheldrick, 2008 ▶); software used to prepare material for publication: *SHELXL97*.

## Supplementary Material

Crystal structure: contains datablock(s) I, global. DOI: 10.1107/S1600536812027432/hb6852sup1.cif

Structure factors: contains datablock(s) I. DOI: 10.1107/S1600536812027432/hb6852Isup2.hkl

Supplementary material file. DOI: 10.1107/S1600536812027432/hb6852Isup3.cml

Additional supplementary materials:  crystallographic information; 3D view; checkCIF report

## Figures and Tables

**Fig. 1.**
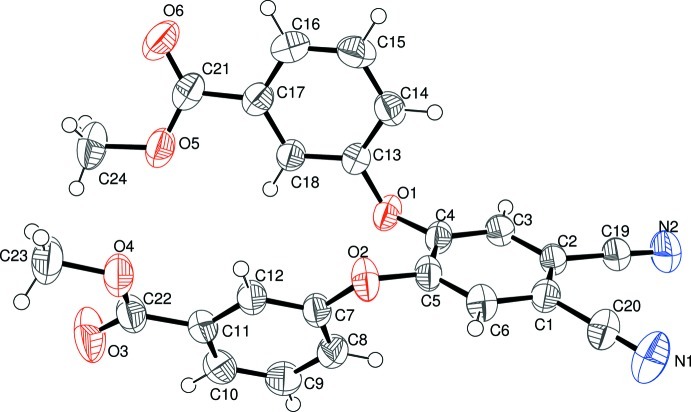
The molecular structure of the title compound. Displacement ellipsoids are drawn at 30% probability level.

**Fig. 2.**
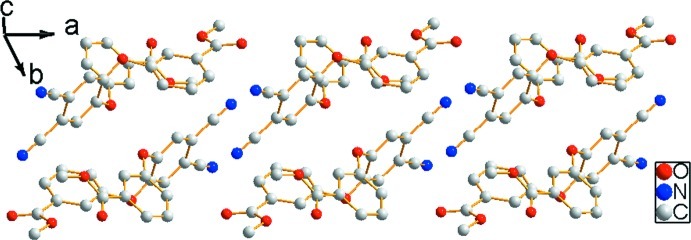
Figure 2. A view of supramolecular configuration of (I).

## supplementary crystallographic information

### Comment

Phthalocyanines are a class of dye and pigments with a wide range of applications. Their macrocyclic basic unit contain four isoindole, which can complex with a range of metal ions. Large rare earth metal ions can bring together these tetrapyrrole derivatives to form sandwich-type double- and triple-decker complexes (Jiang *et al.*, 2009). Depending on the metal centers and the nature of the macrocyclic ligands, these compounds exhibit tunable spectroscopic, electronic, and redox properties, and different extents of intramolecular π-π interactions. Some of the properties of the sandwich-type complexes are unique and enable them to be used as advanced materials for various applications (Wang *et al.*, 2011). As an initial extension of our work on the substituent effect on the phthalocyanine properties, the title compound, as a precursor to synthesize phthalocyanine, was synthesized and characterized by X-ray diffraction, as shown in Fig. 1.

The compound (I) crystallizes in the triclinic system with only two molecule per unit cell, contains one 4,5-dicyano-1,2-phenylene C_8_H_2_N_2_ as main framework and two 3-(methoxycarbonyl)phenolate C_8_H_7_O_3_ substituents, and the C_8_H_2_N_2_ framework is essentially flat, with the maximum deviation from the least-squares mean plane being 0.014 (4) Å. The dihedral angels between the C_8_H_2_N_2_ unit and two benzene planes 3-(methoxycarbonyl)phenolate substituents are 73.62 (5) and 84.08 (6)°, respectively. As shown in Table 1, the C—C and C—O bond lengths within C_8_H_2_N_2_ framework are not clear distinction, indicating the strongly delocalized π-system nature of the 4,5-dicyano-1,2-phenylene framework.

### Experimental

On The basis of report lately(Wang *et al.*, 2009), to a solution of methyl 3-hydroxybenzoate (3.04 g, 0.02 mol) and anhydrous Na_2_CO_3_ (4.20 g, 0.02 mol) in DMF(25 ml) stirred for 30 min, 4,5-dichlorophthalonitrile (0.98 g, 0.01 mol) was added. The resulting mixture was stirred at 60 *^o^*C for 48 h on the basis of TLC monitored. Then the mixture was poured into water (100 ml), and a slightly yellow solid was yielded and isolated by filtration. The crude product was dried in air, yielding dimethyl 3,3'-((4,5-dicyano-1,2-phenylene)bis(oxy))dibenzoate (2.08 g). The solid mixture was chromatographed on a silica gel column using CH_2_Cl_3_/hexane (1:1) as eluent. Repeated chromatography followed by recrystallization from CH_2_Cl_3_ and hexane gave the target compound as colorless blocks. Yield:1.98 g, 46%.

### Refinement

All H atoms were placed in geometrically idealized positions and treated as riding on their parent atoms with C—H = 0.93 Å, *U*_iso_ = 1.2Ueq (C) for aromatic atoms and C—H = 0.96 Å, *U*_iso_ = 1.5Ueq (C) for methyl atoms.

### Crystal data

**Table:**

C_24_H_16_N_2_O_6_	*Z* = 2
*M**_r_* = 428.39	*F*(000) = 444
Triclinic, *P*1	*D*_x_ = 1.404 Mg m^−^^3^
Hall symbol: -P 1	Cu *K*α radiation, λ = 1.54178 Å
*a* = 10.1092 (11) Å	Cell parameters from 3269 reflections
*b* = 10.3408 (11) Å	θ = 2.1–64.7°
*c* = 10.8190 (14) Å	µ = 0.86 mm^−^^1^
α = 82.284 (10)°	*T* = 293 K
β = 85.991 (10)°	Block, colorless
γ = 64.721 (11)°	0.15 × 0.11 × 0.08 mm
*V* = 1013.3 (2) Å^3^	

### Data collection

**Table:**

Agilent Xcalibur Eos Gemini diffractometer	3484 independent reflections
Radiation source: fine-focus sealed tube	2790 reflections with *I* > 2σ(*I*)
Graphite monochromator	*R*_int_ = 0.019
Detector resolution: 0 pixels mm^-1^	θ_max_ = 66.0°, θ_min_ = 4.1°
ω scans	*h* = −11→11
Absorption correction: multi-scan (*CrysAlis PRO*; Agilent, 2011)	*k* = −9→12
*T*_min_ = 0.882, *T*_max_ = 0.935	*l* = −9→12
6668 measured reflections	

### Refinement

**Table:**

Refinement on *F*^2^	Secondary atom site location: difference Fourier map
Least-squares matrix: full	Hydrogen site location: inferred from neighbouring sites
*R*[*F*^2^ > 2σ(*F*^2^)] = 0.039	H-atom parameters constrained
*wR*(*F*^2^) = 0.119	*w* = 1/[σ^2^(*F*_o_^2^) + (0.063*P*)^2^ + 0.0632*P*] where *P* = (*F*_o_^2^ + 2*F*_c_^2^)/3
*S* = 1.07	(Δ/σ)_max_ = 0.010
3484 reflections	Δρ_max_ = 0.21 e Å^−^^3^
292 parameters	Δρ_min_ = −0.20 e Å^−^^3^
1 restraint	Extinction correction: *SHELXL97* (Sheldrick, 2008), Fc^*^=kFc[1+0.001xFc^2^λ^3^/sin(2θ)]^-1/4^
Primary atom site location: structure-invariant direct methods	Extinction coefficient: 0.0036 (6)

### Special details

**Table:**

Experimental. Absorption correction: CrysAlisPro, Agilent Technologies, Version 1.171.35.19 (release 27-10-2011 CrysAlis171 .NET) (compiled Oct 27 2011,15:02:11) Empirical absorption correction using spherical harmonics, implemented in SCALE3 ABSPACK scaling algorithm.
Geometry. All e.s.d.'s (except the e.s.d. in the dihedral angle between two l.s. planes) are estimated using the full covariance matrix. The cell e.s.d.'s are taken into account individually in the estimation of e.s.d.'s in distances, angles and torsion angles; correlations between e.s.d.'s in cell parameters are only used when they are defined by crystal symmetry. An approximate (isotropic) treatment of cell e.s.d.'s is used for estimating e.s.d.'s involving l.s. planes.
Refinement. Refinement of *F*^2^ against ALL reflections. The weighted *R*-factor *wR* and goodness of fit *S* are based on *F*^2^, conventional *R*-factors *R* are based on *F*, with *F* set to zero for negative *F*^2^. The threshold expression of *F*^2^ > σ(*F*^2^) is used only for calculating *R*-factors(gt) *etc*. and is not relevant to the choice of reflections for refinement. *R*-factors based on *F*^2^ are statistically about twice as large as those based on *F*, and *R*- factors based on ALL data will be even larger.

### Fractional atomic coordinates and isotropic or equivalent isotropic displacement parameters (Å^2^)

**Table:**

	*x*	*y*	*z*	*U*_iso_*/*U*_eq_	
O1	0.36679 (12)	0.87260 (13)	0.45190 (11)	0.0500 (3)	
O2	0.53609 (14)	0.59249 (13)	0.39290 (10)	0.0547 (3)	
O3	0.2830 (2)	0.7221 (2)	−0.11360 (13)	0.0933 (6)	
O4	0.26548 (15)	0.56809 (16)	0.04354 (12)	0.0648 (4)	
O5	−0.01163 (14)	0.88074 (16)	0.18060 (12)	0.0635 (4)	
O6	−0.19904 (13)	0.89259 (17)	0.30884 (15)	0.0738 (4)	
N1	1.0057 (2)	0.4624 (2)	0.7376 (2)	0.0809 (6)	
N2	0.7608 (2)	0.8509 (2)	0.83423 (15)	0.0723 (5)	
C1	0.75592 (17)	0.61523 (18)	0.63383 (14)	0.0447 (4)	
C2	0.66927 (17)	0.75363 (18)	0.66704 (13)	0.0430 (4)	
C3	0.53669 (17)	0.83798 (18)	0.60837 (14)	0.0450 (4)	
H3	0.4778	0.9289	0.6315	0.054*	
C4	0.49313 (17)	0.78577 (18)	0.51556 (14)	0.0432 (4)	
C5	0.57990 (18)	0.64856 (18)	0.48157 (14)	0.0449 (4)	
C6	0.71024 (19)	0.56367 (18)	0.54090 (15)	0.0479 (4)	
H6	0.7675	0.4720	0.5187	0.058*	
C7	0.51505 (17)	0.66227 (18)	0.27123 (14)	0.0449 (4)	
C8	0.57881 (19)	0.7533 (2)	0.22567 (16)	0.0514 (4)	
H8	0.6374	0.7728	0.2761	0.062*	
C9	0.5536 (2)	0.8156 (2)	0.10261 (17)	0.0586 (5)	
H9	0.5948	0.8783	0.0706	0.070*	
C10	0.4682 (2)	0.7854 (2)	0.02736 (16)	0.0568 (5)	
H10	0.4523	0.8275	−0.0550	0.068*	
C11	0.40625 (18)	0.69222 (19)	0.07461 (15)	0.0482 (4)	
C12	0.43022 (18)	0.62997 (18)	0.19734 (15)	0.0469 (4)	
H12	0.3894	0.5669	0.2296	0.056*	
C13	0.24755 (16)	0.83783 (17)	0.47464 (14)	0.0416 (4)	
C14	0.21672 (18)	0.78724 (18)	0.59275 (15)	0.0491 (4)	
H14	0.2801	0.7667	0.6583	0.059*	
C15	0.0898 (2)	0.7680 (2)	0.61111 (17)	0.0587 (5)	
H15	0.0671	0.7339	0.6899	0.070*	
C16	−0.00390 (19)	0.7989 (2)	0.51367 (18)	0.0575 (5)	
H16	−0.0905	0.7879	0.5277	0.069*	
C17	0.02986 (17)	0.84627 (18)	0.39509 (16)	0.0469 (4)	
C18	0.15724 (17)	0.86604 (17)	0.37489 (15)	0.0429 (4)	
H18	0.1815	0.8977	0.2957	0.051*	
C19	0.71881 (18)	0.8084 (2)	0.76095 (15)	0.0500 (4)	
C20	0.8951 (2)	0.5287 (2)	0.69212 (18)	0.0556 (4)	
C21	−0.07390 (18)	0.87628 (19)	0.29280 (18)	0.0537 (4)	
C22	0.3129 (2)	0.6638 (2)	−0.00915 (16)	0.0563 (5)	
C23	0.1746 (2)	0.5351 (3)	−0.0331 (2)	0.0756 (6)	
H23A	0.0814	0.6165	−0.0446	0.113*	
H23B	0.1605	0.4533	0.0073	0.113*	
H23C	0.2215	0.5137	−0.1128	0.113*	
C24	−0.1000 (3)	0.8966 (3)	0.0748 (2)	0.0795 (7)	
H24A	−0.1324	0.8210	0.0842	0.119*	
H24B	−0.0428	0.8913	−0.0006	0.119*	
H24C	−0.1834	0.9882	0.0706	0.119*	

### Atomic displacement parameters (Å^2^)

**Table:**

	*U*^11^	*U*^22^	*U*^33^	*U*^12^	*U*^13^	*U*^23^
O1	0.0467 (6)	0.0577 (7)	0.0502 (6)	−0.0286 (6)	−0.0169 (5)	0.0096 (5)
O2	0.0801 (9)	0.0585 (7)	0.0403 (6)	−0.0430 (7)	−0.0184 (5)	0.0024 (5)
O3	0.1357 (15)	0.1149 (14)	0.0484 (8)	−0.0717 (12)	−0.0370 (9)	0.0116 (8)
O4	0.0720 (9)	0.0846 (10)	0.0503 (7)	−0.0426 (8)	−0.0150 (6)	−0.0085 (7)
O5	0.0553 (7)	0.0812 (9)	0.0595 (8)	−0.0331 (7)	−0.0194 (6)	−0.0020 (6)
O6	0.0421 (7)	0.0856 (10)	0.0996 (11)	−0.0272 (7)	−0.0076 (7)	−0.0257 (8)
N1	0.0627 (11)	0.0727 (12)	0.1003 (14)	−0.0196 (9)	−0.0356 (10)	0.0000 (10)
N2	0.0790 (12)	0.0964 (14)	0.0541 (9)	−0.0447 (11)	−0.0119 (8)	−0.0180 (9)
C1	0.0451 (9)	0.0530 (9)	0.0404 (8)	−0.0258 (8)	−0.0071 (6)	0.0011 (7)
C2	0.0454 (8)	0.0566 (9)	0.0331 (7)	−0.0273 (7)	−0.0041 (6)	−0.0034 (6)
C3	0.0462 (9)	0.0517 (9)	0.0400 (8)	−0.0233 (7)	−0.0017 (6)	−0.0052 (7)
C4	0.0443 (8)	0.0520 (9)	0.0374 (7)	−0.0257 (7)	−0.0096 (6)	0.0040 (6)
C5	0.0548 (9)	0.0540 (9)	0.0363 (8)	−0.0332 (8)	−0.0091 (7)	0.0003 (7)
C6	0.0546 (10)	0.0472 (9)	0.0444 (9)	−0.0230 (8)	−0.0073 (7)	−0.0047 (7)
C7	0.0479 (9)	0.0506 (9)	0.0374 (8)	−0.0214 (7)	−0.0064 (6)	−0.0035 (7)
C8	0.0511 (9)	0.0613 (10)	0.0504 (9)	−0.0318 (8)	−0.0033 (7)	−0.0063 (8)
C9	0.0630 (11)	0.0674 (12)	0.0525 (10)	−0.0370 (10)	0.0042 (8)	−0.0002 (8)
C10	0.0604 (11)	0.0681 (12)	0.0402 (9)	−0.0276 (9)	−0.0014 (7)	0.0012 (8)
C11	0.0484 (9)	0.0570 (10)	0.0386 (8)	−0.0209 (8)	−0.0031 (7)	−0.0067 (7)
C12	0.0512 (9)	0.0541 (9)	0.0403 (8)	−0.0265 (8)	−0.0048 (7)	−0.0050 (7)
C13	0.0391 (8)	0.0427 (8)	0.0440 (8)	−0.0178 (7)	−0.0048 (6)	−0.0047 (6)
C14	0.0530 (9)	0.0520 (10)	0.0395 (8)	−0.0195 (8)	−0.0034 (7)	−0.0040 (7)
C15	0.0596 (11)	0.0651 (11)	0.0501 (10)	−0.0279 (9)	0.0105 (8)	−0.0031 (8)
C16	0.0458 (9)	0.0620 (11)	0.0661 (11)	−0.0254 (9)	0.0077 (8)	−0.0076 (9)
C17	0.0407 (8)	0.0447 (9)	0.0557 (10)	−0.0169 (7)	−0.0034 (7)	−0.0094 (7)
C18	0.0418 (8)	0.0459 (8)	0.0423 (8)	−0.0199 (7)	−0.0046 (6)	−0.0030 (6)
C19	0.0508 (9)	0.0649 (11)	0.0396 (8)	−0.0286 (9)	−0.0041 (7)	−0.0064 (7)
C20	0.0548 (11)	0.0584 (11)	0.0580 (10)	−0.0272 (9)	−0.0146 (8)	−0.0024 (8)
C21	0.0429 (9)	0.0481 (9)	0.0723 (12)	−0.0185 (8)	−0.0101 (8)	−0.0124 (8)
C22	0.0604 (11)	0.0652 (11)	0.0419 (9)	−0.0232 (9)	−0.0091 (8)	−0.0087 (8)
C23	0.0767 (14)	0.0902 (16)	0.0725 (13)	−0.0414 (13)	−0.0213 (11)	−0.0192 (12)
C24	0.0740 (14)	0.0946 (17)	0.0736 (14)	−0.0367 (13)	−0.0357 (11)	−0.0003 (12)

### Geometric parameters (Å, º)

**Table:**

O1—C4	1.3746 (19)	C8—H8	0.9300
O1—C13	1.3956 (19)	C9—C10	1.379 (3)
O2—C5	1.3714 (19)	C9—H9	0.9300
O2—C7	1.3970 (19)	C10—C11	1.386 (3)
O3—C22	1.200 (2)	C10—H10	0.9300
O4—C22	1.323 (2)	C11—C12	1.385 (2)
O4—C23	1.449 (2)	C11—C22	1.491 (2)
O5—C21	1.332 (2)	C12—H12	0.9300
O5—C24	1.450 (2)	C13—C14	1.381 (2)
O6—C21	1.206 (2)	C13—C18	1.382 (2)
N1—C20	1.138 (2)	C14—C15	1.378 (3)
N2—C19	1.141 (2)	C14—H14	0.9300
C1—C6	1.388 (2)	C15—C16	1.379 (3)
C1—C2	1.403 (2)	C15—H15	0.9300
C1—C20	1.440 (2)	C16—C17	1.384 (3)
C2—C3	1.390 (2)	C16—H16	0.9300
C2—C19	1.440 (2)	C17—C18	1.386 (2)
C3—C4	1.380 (2)	C17—C21	1.488 (2)
C3—H3	0.9300	C18—H18	0.9300
C4—C5	1.396 (2)	C23—H23A	0.9600
C5—C6	1.378 (2)	C23—H23B	0.9600
C6—H6	0.9300	C23—H23C	0.9600
C7—C8	1.376 (2)	C24—H24A	0.9600
C7—C12	1.378 (2)	C24—H24B	0.9600
C8—C9	1.389 (3)	C24—H24C	0.9600
			
C4—O1—C13	117.31 (12)	C11—C12—H12	120.3
C5—O2—C7	118.67 (13)	C14—C13—C18	122.10 (15)
C22—O4—C23	115.98 (16)	C14—C13—O1	121.36 (14)
C21—O5—C24	116.20 (16)	C18—C13—O1	116.39 (13)
C6—C1—C2	119.92 (15)	C15—C14—C13	118.36 (16)
C6—C1—C20	119.81 (16)	C15—C14—H14	120.8
C2—C1—C20	120.21 (15)	C13—C14—H14	120.8
C3—C2—C1	119.98 (14)	C14—C15—C16	120.56 (16)
C3—C2—C19	120.20 (15)	C14—C15—H15	119.7
C1—C2—C19	119.82 (15)	C16—C15—H15	119.7
C4—C3—C2	119.47 (16)	C15—C16—C17	120.55 (17)
C4—C3—H3	120.3	C15—C16—H16	119.7
C2—C3—H3	120.3	C17—C16—H16	119.7
O1—C4—C3	119.52 (15)	C16—C17—C18	119.65 (16)
O1—C4—C5	119.78 (14)	C16—C17—C21	118.46 (16)
C3—C4—C5	120.62 (15)	C18—C17—C21	121.89 (16)
O2—C5—C6	118.30 (15)	C13—C18—C17	118.74 (15)
O2—C5—C4	121.55 (15)	C13—C18—H18	120.6
C6—C5—C4	120.09 (15)	C17—C18—H18	120.6
C5—C6—C1	119.90 (16)	N2—C19—C2	178.62 (18)
C5—C6—H6	120.0	N1—C20—C1	178.7 (2)
C1—C6—H6	120.0	O6—C21—O5	123.65 (17)
C8—C7—C12	121.66 (15)	O6—C21—C17	124.32 (18)
C8—C7—O2	122.68 (14)	O5—C21—C17	112.02 (14)
C12—C7—O2	115.61 (15)	O3—C22—O4	123.13 (18)
C7—C8—C9	118.44 (16)	O3—C22—C11	123.71 (19)
C7—C8—H8	120.8	O4—C22—C11	113.16 (15)
C9—C8—H8	120.8	O4—C23—H23A	109.5
C10—C9—C8	120.78 (18)	O4—C23—H23B	109.5
C10—C9—H9	119.6	H23A—C23—H23B	109.5
C8—C9—H9	119.6	O4—C23—H23C	109.5
C9—C10—C11	119.90 (16)	H23A—C23—H23C	109.5
C9—C10—H10	120.0	H23B—C23—H23C	109.5
C11—C10—H10	120.0	O5—C24—H24A	109.5
C12—C11—C10	119.81 (16)	O5—C24—H24B	109.5
C12—C11—C22	121.87 (16)	H24A—C24—H24B	109.5
C10—C11—C22	118.31 (16)	O5—C24—H24C	109.5
C7—C12—C11	119.39 (16)	H24A—C24—H24C	109.5
C7—C12—H12	120.3	H24B—C24—H24C	109.5
